# Evaluation of Virulence in Cynomolgus Macaques Using a Virus Preparation Enriched for the Extracellular Form of Monkeypox Virus

**DOI:** 10.3390/v14091993

**Published:** 2022-09-09

**Authors:** Eric M. Mucker, Josh D. Shamblin, Arthur J. Goff, Todd M. Bell, Christopher Reed, Nancy A. Twenhafel, Jennifer Chapman, Marc Mattix, Derron Alves, Robert F. Garry, Lisa E. Hensley

**Affiliations:** 1 United States Army Medical Research Institute of Infectious Diseases, Virology Division, Fort Detrick, Frederick, MD 21702, USA; 2 United States Army Medical Research Institute of Infectious Diseases, Pathology Division, Fort Detrick, Frederick, MD 21702, USA; 3 National Institutes of Health, National Institute of Allergy and Infectious Diseases, Infectious Disease Pathogenesis Section, Rockville, MD 20852, USA; 4 Department of Microbiology and Immunology, School of Medicine, Tulane University, New Orleans, LA 70112, USA; 5 Zalgen Labs, Frederick, MD 21703, USA; 6 Global Virus Network (GVN), Baltimore, MD 21201, USA; 7 United States Department of Agriculture, Zoonotic and Emerging Disease Unit, Manhattan, KS 66505, USA

**Keywords:** orthopoxvirus, extracellular virion, nonhuman primates, viremia, model, smallpox, monkeypox, countermeasures, transmission, dissemination, morphogenesis

## Abstract

The 2022 global human monkeypox outbreak emphasizes the importance of maintaining poxvirus research, including enriching a basic understanding of animal models for developing and advancing therapeutics and vaccines. Intravenous administration of monkeypox virus in macaques is arguably one of the best animal models for evaluating the efficacy of medical countermeasures. Here we addressed one criticism of the model, a requirement for a high-titer administration of virus, as well as improving our understanding of monkeypox virus pathogenesis. To do so, we infected macaques with a challenge dose containing a characterized inoculum enriched for the extracellular form of monkeypox virus. Although there were some differences between diseases caused by the enriched preparation compared with a relatively similar unpurified preparation, we were unable to reduce the viral input with the enriched preparation and maintain severe disease. We found that inherent factors contained within the serum of nonhuman primate blood affect the stability of the monkeypox extracellular virions. As a first step to study a role of the extracellular form in transmission, we also showed the presence of this form in the oropharyngeal swabs from nonhuman primates exposed to monkeypox virus.

## 1. Introduction

Although many have long viewed monkeypox as an emerging threat, monkeypox models have had the main initiative of providing evidence of efficacy for licensing medical countermeasures for smallpox. With the exception of Tembexia™, therapeutics have utilized the intravenous monkeypox virus (MPXV) nonhuman primate (NHP) model to help support licensure. In light of the global 2022 monkeypox outbreak, the screening and subsequent approval of medical countermeasures using the NHP model have proven to be of great benefit. That is, evidence from NHP studies supporting the licensure of smallpox countermeasures can concomitantly provide credence for the treatment of human monkeypox [1]. Unlike other surrogate orthopox viruses, monkeypox virus infection can lead to a systemic rash and fulminant disease in both humans and NHPs. The intravenous monkeypox virus model in macaques, otherwise known as the lesional model, is thought to recapitulate the secondary viremia observed in human systemic smallpox disease [2]. This viremia is responsible for seeding tissues and leads to the formation of mucosal and cutaneous lesions. Although nonhuman primate hosts [3,4] and routes of virus administration [4,5,6,7] have been studied to improve upon the model, especially to reduce the high dose required for severe disease and via a natural route, the nature of the secondary viremia and how it relates to the lesional model have never been explored.

There are two major morphogenic forms of poxviruses, the mature virion (MV) and the enveloped virion (EV) [8]. EVs have an additional membrane relative to MVs and appear antigenically distinct to the host, as the MV proteins are concealed by the extra membrane. EVs that are released from the cell are termed extracellular enveloped virions (EEV) and those retained on the cell surface are termed cell-associated enveloped virions (CEV). Differences between CEVs and EEVs have been described [9], but, given the similarity (and for simplicity), we will use the term EV to represent CEV and EEV. Traditionally, monkeypox virus used for challenge was propagated, collected from cell culture, sonicated, and freeze/thawed to liberate cell-associated virus (MV). As a consequence, the outer membrane of EV within the inoculum is disrupted, leaving only MV.

The in vivo role of EV in the pathogenesis of monkeypox is poorly defined, but there are multiple biological properties described for EV that likely contribute to its in vivo function. These properties include antibody and complement resistance, and relatively faster binding and entry kinetics than MV [10,11,12,13], and could give EV a select advantage in dissemination or transmission [14]. Moreover, the requirement for host responses to EV for protection [2,15,16,17,18] and the success of Siga’s Tecovirimat further suggests a major role in vivo. In vitro, the compound reduces EV by 10-fold, but does not impact the formation or infectivity of MV [19]. Although the efficacy and proposed mechanism for Tecovirimat suggest EV is important for disease, how a decrease in EV production interferes with viral pathogenesis is still unknown.

The exact role of EV in the pathogenesis and transmission of orthopoxviruses is still unknown. By analyzing plasma samples generated from immunocompromised rabbits infected with vaccinia virus, strain IHDJ, Payne proposed a role of EV in dissemination [20]. It is important to note that these studies have not been repeated using immunocompetent hosts and/or a member of the orthopox genus that has greater implications for humans. Furthermore, the quantity of sample needed to repeat these studies is limiting and involves the exsanguination of the host. Studies by Blasco and Moss also provided in vitro evidence for EEV being responsible for distant spread and CEV for cell-to-cell spread [21]. Some postulate that infected macrophages may be responsible for systemic dissemination within a host [22,23], but these and similar questions remain unanswered.

There has also been evidence that EV could be important in transmission. Payne and Kristensson provided evidence for the presence of EV as the major form of virus in the oropharyngeal region of mice infected via intranasal instillation with cowpox virus [24]. Despite the fact that the major extracellular morphogenic form was EV, these data are normally applied to local dissemination and not transmission.

Here, we described a characterized preparation of EV enriched monkeypox virus by its morphogenic composition. We compared the EV enriched preparation to a common preparation of monkeypox virus Zaire (unpurified) given at lethal and semi-lethal doses. We found that the new preparation may have had little, if any impact on disease, and did not affect lethality. We also found that the EVs within our preparation were not stable when administered intravenously. As a primer towards transmission research, we also present data for EV being present in the oral swabs of MPXV-exposed NHPs by electron microscopy.

## 2. Materials and Methods

### 2.1. Viruses, Cells, and Assays

#### 2.1.1. Virus and Propagation

Monkeypox virus strain Zaire was propagated from scab material on chorioallantoic membranes, followed by one passage in LLC-MK2 cells, two passages in BSC-40 cells, and two passages in Vero E6 cells. This material was received from the Centers for Disease Control and Prevention (CDC) and passed in MA104 cells. This virus will be referred to as the master stock and was provided by John Huggins (USAMRIID).

The master stock of virus was thawed, sonicated, and vortexed four times. MA104 cells were inoculated at a multiplicity of infection (MOI) of 0.5 (5 plaque forming units [pfu] for every 10 cells). The flasks were incubated in a 37 °C incubator and rocked every 15 min for 1 h. DMEM supplemented with 5% fetal bovine serum was added to each flask, after which the flasks were returned to the incubator. After 3 days, the cells were monitored for cytopathic effect (CPE) daily. One day after CPE was observed in 100% of the monolayer, the supernatant was removed, cells were scraped and pooled, and three freeze thaw cycles were performed. The material was then subjected to three sonication/vortex cycles and centrifuged to remove cells and larger cell debris. The supernatant was collected and aliquots were made into pre-labeled tubes. This material was the “crude” or “unpurified” working stock. A portion of this material was purified via a sucrose cushion, followed by a sucrose gradient [25] and was provided by Lisa Hensley (USAMRIID).

Before exposing the animals to a fresh preparation of monkeypox virus, the conditions were defined. The dose response for the intravenous model is quite steep and relatively small differences in titer could equate to more severe disease [26,27]. The goal was to obtain a titer of approximately 5 × 10^6^ pfu/mL, (approximately 1× LD50 [28]) so that increases in virulence with the EV enriched inoculum could be assessed. Inoculums of 5 × 10^7^ pfu/mL (or greater) would be less useful as it is a dose typically used to evaluate fulminant disease in the lesional MPXV NHP models. Therefore, multiple conditions were utilized to maximize the titer of the enriched virus stock.

Fresh MPXV for the enriched EV preparations was produced by infecting Vero E6 cells (approx. 2 × 10^7^ cells/flask) with crude working stock MPXV. The virus was adsorbed for 1 h, monolayers washed 4× with warmed EMEM with 2% heat-inactivated serum and provided a liquid overlay of the same media (10 mL). The supernatant was collected and clarified (removal of cell/cell debris) by centrifugation (1000× *g* for 10 min at 4 °C). For all preparations, with the exception of the EV characterization experiments, an MOI of 30 and a harvest time of 24 h were used. So that only freshly prepared virus was used, this process was repeated for each experiment.

#### 2.1.2. Virus Titrations

Monkeypox virus, to include the purified and crude inoculums, was titrated using a standard plaque assay. One hundred microliters of sample was adsorbed onto at least 2 wells containing Vero-E6 cells (6-well dishes) for 1 h in a 37 °C incubator, with gentle rocking every 15 min. Two milliliters of EMEM containing 2% heat-inactivated FBS was added to each well and allowed to incubate for 4 days at 37 °C. The wells were then stained with a gentian violet solution (0.4% gentian violet) for at least 30 min, after which the stain was removed and the monolayers washed with distilled water. Plaques were subsequently enumerated.

Freshly prepared stocks would have to be used to avoid issues with EV integrity. We first had to define a methodology to both understand the morphogenic makeup and also the repeatability of our virus preparation prior to animal inoculation. A fresh virus was prepared 24 h prior to intravenous exposure implementing the aforementioned conditions. Since the route of exposure was intravenous, we tested whether or not the susceptibility of the virions to MV-neutralizing antibody would change after the mechanical stress of expunging the material through a syringe (i.e., shearing EV membrane). For the fresh preparation (EV) inoculums, the supernatant was tested by plaque assay by pipetting or by expunging through a 1 mL syringe, in the presence or absence (PBS alone) of a 1:50 dilution of MAb-7D11 (anti-MV antibody).

#### 2.1.3. Fresh (EV) Characterization

Because the EV inoculum would have to be prepared from a fresh propagation on the day of challenge, initial experiments were conducted to ascertain the quantity of total virus, repeatability, and presence and relative quantity of EV. MPXV EV was propagated as stated above (viruses and cells) at an MOI of 3 and 30, and harvested at two time points, 24 and 48 h. The collected material was treated with either PBS or anti-MV antibody, MAb-7D11 and incubated for 30 min at 37 °C and plaque titrated.

#### 2.1.4. MV Neutralization

Although it has been reported that EVs are relatively resistant to the effects of serum that is homologous with the species from which the virions were propagated, we sought to confirm that intact EV could be responsible for potential differences observed by examining the susceptibility of monkeypox virus EV to NHP serum. To do so, MV neutralization assays and comet inhibition assays (Section 2.1.5) were conducted. For MV neutralization assays, clarified fresh preparations or crude MPXV was spiked (1:1) into cynomolgus or African green macaque (AGM) heat-inactivated serum (HI), or media (EMEM with 2% heat-inactivated FBS) and incubated with either 7D11, 10F5, or PBS (pH 7.4). The spiked media, serum, and heat-inactivated serum were split into two groups, one that was subject to four freeze/thaw and sonication cycles and one that was not manipulated. Dilutions of the antibody or PBS were added (1:1). All the samples containing PBS or antibody were incubated for 30 min at 37 °C and subsequently plaque titrated as described. Anti-L1 antibodies, 10F5 and 7D11, were provided by Jay Hooper (USAMRIID). Statistical comparisons of percent neutralization between all groups were made using a Two-way Analysis of Variance and Bonferoni multiple comparisons using Graphpad Prism.

#### 2.1.5. Comet Inhibition Assays

Monkeypox virus was adsorbed to Vero E6 cells for 2 h before washing four times with warmed (37 °C) PBS. The wells were subsequently overlayed with EMEM containing 10% of either HI FBS, cynomolgus macaque serum, HI cynomolgus macaque serum, AGM serum, or HI AGM serum. Before addition to the well, 7D11 (1:500) or an equal volume of PBS was added to each. As a no comet control, 1.5% methylcellulose (final concentration) was also used. Plates were placed in a 37 °C incubator for 5 days at an approximate angle of 10 degrees. Plates were removed, stained with crystal violet, and washed with PBS. Pictures were acquired and qualitatively evaluated.

#### 2.1.6. Transmission Immunoelectron Microscopy

We questioned whether EV could potentially play a role in respiratory transmission by confirming its existence in oral/throat samples by IEM. To do so, stocks and oropharyngeal swabs were diluted 1:1 with PBS, added to grids, absorbed for 20 min, and washed three times with PBS. The grids were subsequently blocked for 20 min using 4% PBS-buffered normal goat serum. A 1:100 dilution of either MAb-5D8 (anti-MV) or MAb-10F10 (anti-EV), primary antibodies (provided by Jay Hooper, USAMRIID), was added, incubated for 45–60 min, and washed three times with PBS. This procedure was repeated for the application of the 1:40 secondary antibody (gold-conjugated). The grids were then fixed in 2% glutaraldehyde for 20 min and washed three times using dH_2_O, followed by at least a 1 h exposure to 1% osmium tetroxide fumes before removal from Biosafety Level 3. Grids were then rinsed with distilled water five times and examined using a JEOLJEM-1011 transmission electron microscope.

### 2.2. Nonhuman Primates

#### 2.2.1. Conduct and Exposure

Adult, male Mauritius cynomolgus macaques (*Macaca fascicularis*) were screened for neutralizing antibodies to monkeypox virus prior to infection as described below. We opted to use cynomolgus macaques since they tend to exhibit more severe disease than rhesus macaques and, as such, are more sensitive to the virus [27]. Macaques were exposed using the EV enriched preparation and compared to macaques (n = 3 per group) exposed using our typical preparation [29]. Correlates specific to the intravenous model were captured and compared. This study was powered for evaluating statistical differences in lethality. A second study would have been performed to statistically resolve potential differences in lethality or other correlates of disease.

Physical examinations, lesion counts, and blood draws were performed prior to challenge, the day of challenge, and every two days post-challenge until day 14. After which, day 17 and day 20 data (termination of the study) were collected. Blood samples were collected via the femoral vein for all bleeds. Aliquots of EDTA blood were placed in a −80 °C freezer. Serum tubes were allowed to clot, centrifuged and serum removed according to manufacturer’s instructions. Serum was immediately utilized for blood chemistries. All exposures were performed by intravenous infusion via the saphenous vein.

An extra set of oropharyngeal swabs were collected from animals (unvaccinated) reported in Golden et al. [30], placed in PBS, centrifuged at 1000× *g* for 10 min, and supernatants removed before processing for IEM. All samples were freshly prepared. Postmortem examinations were performed by a board-certified veterinary pathologist in USAMRIID’s Veterinary Pathology Division.

#### 2.2.2. NHP Neutralization Assays (Prescreening)

To confirm animals were not previously exposed to orthopox viruses, neutralization assays were performed. Serum from animals was heat-inactivated (56 °C for 30 min) and serially diluted in MEM alpha with 2% heat-inactivated FBS and HEPES. A target of 100 pfu/100 uL was added to each dilution and allowed to incubate at 4 °C overnight. Both positive (vaccinia immunoglobulin) and negative (media only) controls were concomitantly prepared. Titration of the samples was performed in duplicate wells by plaque assay. Results were reported as a percent reduction of the negative control.

#### 2.2.3. DNA Extraction and Quantitative PCR (QPCR)

Extractions and QPCR were performed as described previously [31]. Briefly, 100 µL of whole EDTA whole blood was extracted using Qiagen DNA Blood Kit and eluted using 100 µL of buffer provided by the manufacturer. For QPCR, 5 µL of the eluted DNA was assayed in duplicate using the assay described in Kulesh et al. [32]. The averaged values were multiplied by 200 to yield genomes per milliliter. When averaged replicates fell below the limit of detection, data were stated as the limit of detection (2.5 copies/5 uL).

#### 2.2.4. Chemistry and Hematology

Hematological data were generated on an ACT 10 Beckmann Coulter using whole EDTA blood. Abaxis Piccolos were used to evaluate clinical chemistries using Abaxis Chem12 or Chem13 reagent disks using serum samples.

#### 2.2.5. Postmortem Examination, Histology, and Immunohistochemistry (IHC)

A postmortem examination (i.e., necropsy) was performed on all animals, either as soon as death occurred or after euthanasia of terminally ill or moribund animals. All tissues were immersion-fixed in 10% neutral buffered formalin for a minimum of 21 days, according to Institute protocol.

Formalin-fixed tissues for histologic examination were trimmed, processed, and embedded in paraffin according to established protocols [33]. Histology sections were cut at 5 µm, mounted on glass slides, and stained with hematoxylin and eosin (H&E). Immunohistochemical staining was performed on replicate tissues sections using an Envision + kit (DAKO, Carpinteria, CA, USA). Normal tissue served as the negative control; the positive control was from a known monkeypox virus-infected nonhuman primate; and normal (uninfected) IgG was used as the negative control. Briefly, sections were deparaffinized in xyless II (Val Tech Diagnostics Inc., Brakenridge, USA), rehydrated in graded ethanol, and endogenous peroxidase activity was quenched in a 0.3% hydrogen peroxide/methanol solution for 30 min at room temperature. Slides were washed in PBS then sections were incubated in the primary antibody, a non-commercial rabbit polyclonal antibody against vaccinia virus, diluted 1:3500 for 60 min at room temperature. Sections were washed in PBS and incubated for 30 min with Envision + rabbit secondary reagent (horseradish peroxidase-labeled polymer) at room temperature. Peroxidase activity was developed with 3,3′-diaminobenzidine (DAB), counterstained with hematoxylin, dehydrated, cleared with xyless II (Val Tech Diagnostics Inc., Brakenridge, USA), then coverslipped.

## 3. Results

### 3.1. Virus Preparation and Characterization

The act of freezing/thawing and/or sonication of fresh preparations of vaccinia virus will destroy the integrity of the outer envelope of EV [34]. To test whether or not the crude preparation of monkeypox virus, which had been previously subjected to 4 rounds of freeze/thaw and sonication, contained EV, we examined the crude material by immunoelectron microscopy using antibodies specific for the outer envelope protein A33 (MAb-10F10) [35] or the MV protein D8 (MAb-5D8) [36]. Virions were consistent with MV particles and were tagged (4+) when anti-D8 antibody was present and little or no tagging in the presence of the anti-A33 antibody. To confirm this finding, neutralizing assays were performed in the presence of anti-MV antibody, MAb-7D11 (presented in later sections).

**Figure 1:**
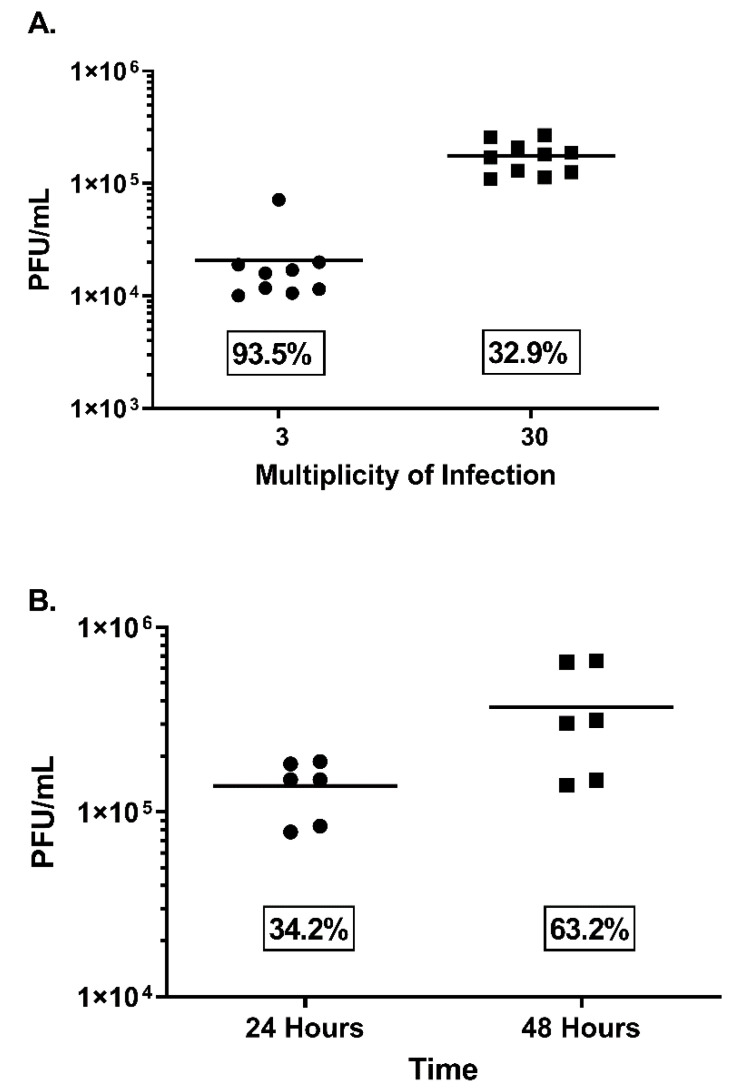
Evaluation of fresh preparations of monkeypox virus from supernatants using different multiplicities of infections and harvest times. High-multiplicity of infections (3 and 30 pfu/cell) were utilized for synchronization of viral infection on confluent Vero E6 cells plated in T175 flasks. Three experiments were conducted for each MOI (**A**) and time point (**B**). Titers are given (PFU/mL) as well as variability, as determined by % coefficient of variation (CV), and are shown in text boxes on the graph.

**Figure 2:**
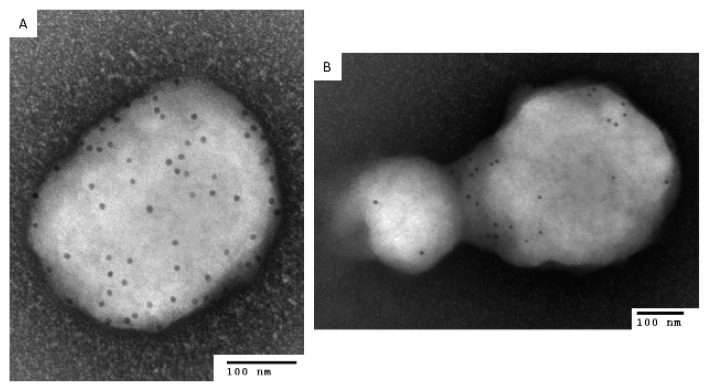
Immunoelectron microscopy of a fresh preparation of monkeypox virus using either an anti-EV antibody (10F10) or anti-MV antibody (5D8). To confirm the relative absence or presence of EV in our crude and/or fresh preparations of monkeypox virus, immunoelectron microscopy was utilized. (**A**) Crude preparation with anti-MV antibody; (**B**) fresh preparation with anti-EV antibody. Scale bars are 100 nM.

Initially, two MOIs were tested (3 and 30) to evaluate total virus output (Figure 1A). An MOI of 30 provided approximately one log more virus, a mean of 1.8 × 10^5^ versus 2.1 × 10^4^ pfu/mL, in 24 h with limited CPE (≤1) and a tighter coefficient of variation (CV) (Figure 1A). Using this MOI, an incubation time of 48 h was assessed (Figure 1B). Again, the 24 h incubation produced similar results (1.2 × 10^5^ pfu/mL), whereas the 48 h incubation produced similar virus in the supernatant (6.6 × 10^5^ pfu/mL) with more CPE (≥2) and a greater % CV (Figure 1B). To provide evidence for the presence of EV under these conditions, a two-pronged approach was used. Initially, anti-MV antibody (7D11) or PBS was incubated with a portion of the supernatants and plaque titrated. The antibody neutralized all but 11% of the virus, whereas after freeze/thaw, there was 97.9% neutralization (n = 3). It is important to note that 100% neutralization of the crude stock (or disrupted fresh material) was not obtained, even when antibody concentration was increased, or virus input was decreased (data not shown). Therefore, differences in neutralization representing EV content are approximate and based on differences from the control (freeze/thaw). In separate experiments, under the same conditions, the supernatant was subjected to immunoelectron microscopy using either the anti-EV envelope antibody to A33, 10F10 or the anti-MV envelope protein to D8, 5B8. In all cases, both tagged and untagged virions were documented, owing to the presence of both morphological entities (Figure 2). From this and the neutralization data we can confirm that EVs were present in the preparation and that they comprised, at most, 8.3% of the infectious virus contained within. Because of the repeatability, acceptable titer, and the presence of EV, we utilized these conditions for the preparation of the NHP challenge material.

### 3.2. Preparation of Inoculum

**Figure 3:**
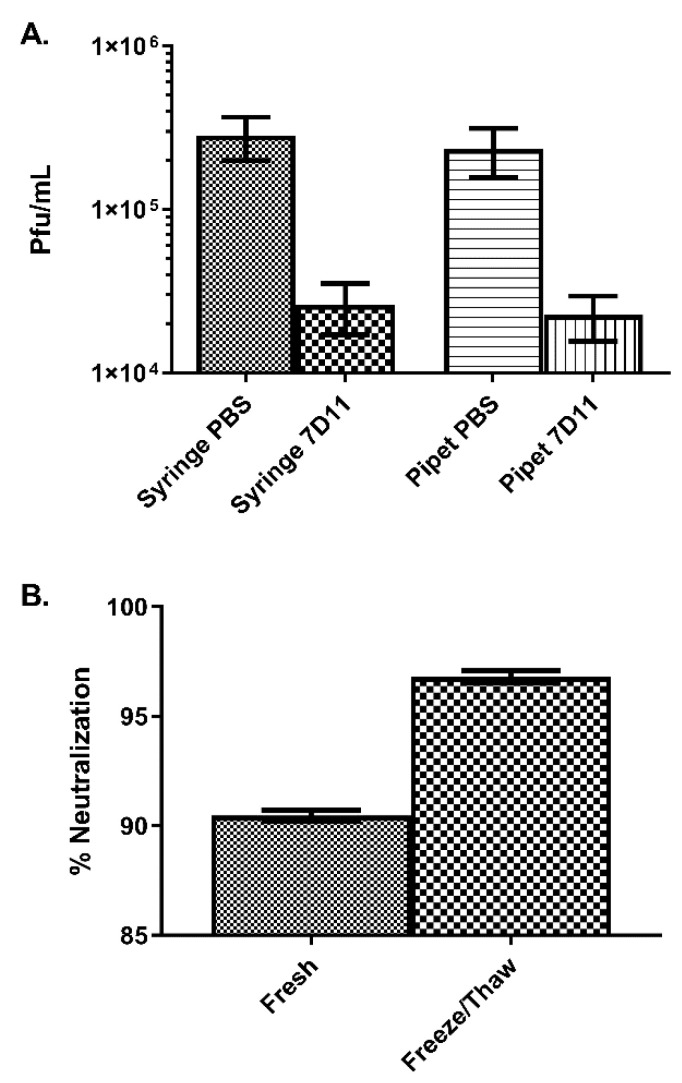
Titration of inoculum in the presence or absence of anti-MV antibody. (**A**) Back titration of the inoculum was performed by either pipetting or expunging the inoculum through a syringe subsequent to plaque assay. Antibody known to neutralize the MV form of monkeypox virus (7D11) was used to evaluate the morphogenic content (EV and MV). (**B**) The inoculums subsequently underwent four sequences of a freeze/thaw and three sonication cycles, after which a neutralization assay was performed.

Samples were prepared from the supernatant using a syringe or pipet and treated with either PBS or 7D11 (1:50). Both the syringe and pipet PBS titers for the inoculum were similar (2.8 × 10^5^ pfu/mL and 2.4 × 10^5^ pfu/mL, respectively). The syringe and pipet samples containing MV-neutralizing antibody were also quite similar, 2.6 × 10^4^ pfu/mL and 2.3 × 10^4^ pfu/mL, respectively, with a total neutralization of 90.2% and 90.7%, (Figure 3A). To ensure that there was a particle protected from specific antibody neutralization and to calculate the EV content, the samples were freeze/thawed and sonicated to disrupt the outer membrane [34]. Neutralization in the disrupted material increased to 96.8% without a change in overall titer (2.9 × 10^5^ pfu/mL), alluding to a maximum EV content of approximately 6.6% (Figure 3B). This equates to a dose of 1.8 × 10^4^ pfu/mL of antibody resistant EV.

### 3.3. Challenge of NHP with an Enriched EV Preparation

Data from animals intravenously inoculated with crude preparation of MPXV at a uniformly lethal (5 × 10^7^ pfu/mL) and partially lethal (5 × 10^6^ pfu/mL) dose are included for comparison. More details of this research will be published elsewhere [29]. Direct comparisons were made with the lethal model, as our goal was to lower the dose required to produce a similar mortality.

#### 3.3.1. Survival

**Figure 4:**
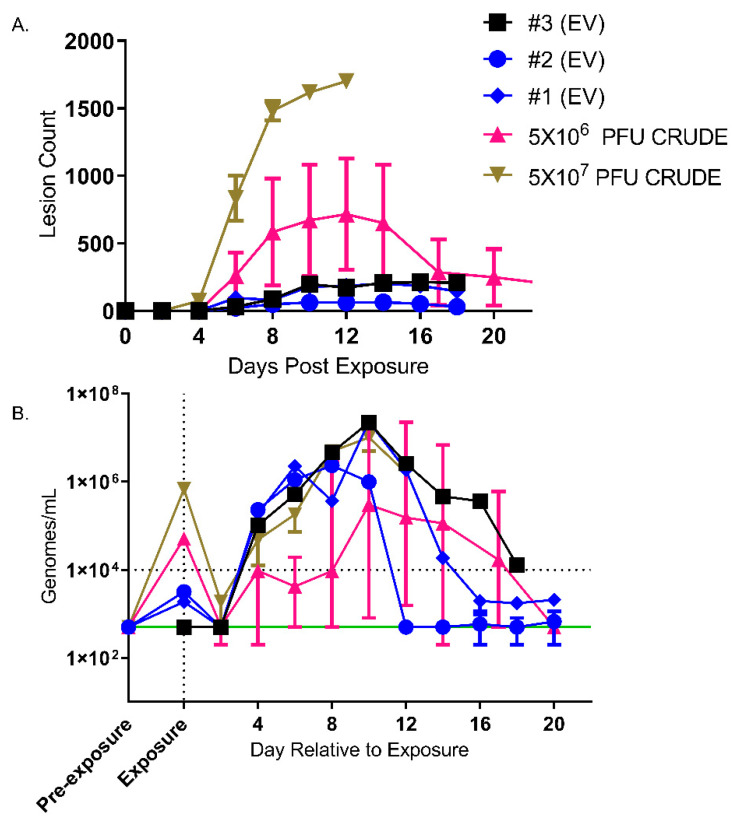
Assessment of lesion burden and QPCR. Lesions (**A**), viremia (QPCR) (**B**) over time are shown. Mean with standard error of the mean is shown for lesion counts (**A**) and median and error are shown for QPCR data (**B**). Animal #3 (EV) was shown to have extravasation of the challenge inoculum and is shown in black (square symbol). Green line, lower limit of detection. Horizontal dotted line, lower limit of quantitation. Blood obtained previous to exposure is shown on the *X*-axis as “Pre-exposure”. Blood obtained within 2 min of intravenous exposure was on Day 0 and is shown on the *X*-axis as “Exposure”.

Three animals were intravenously exposed to 1.8 × 10^4^ pfu of MPXV EV (2.8 × 10^5^ total pfu). In all three NHPs, an attenuated disease course, as compared to the 5 × 10^6^ pfu/mL or 5 × 10^7^ pfu/mL IV model, was noted (Figure 4). More specifically, no animals succumbed to typical disease, although one animal was humanely euthanized due to necrosis of the hind limb, secondary to inadvertent iatrogenic introduction of MPXV EV into the adjacent skeletal muscle upon intravenous challenge. This animal was not considered moribund due to MPXV disease but was humanely euthanized due to declining clinical condition.

#### 3.3.2. Disease Development, Viral and Lesion Burden

The disease course in the animals exposed to the EV enriched preparation was very mild. Lymphadenopathy was noted by day 6 in all animals. Papules were present on day 6 and progressed in a typical manner: papule, vesicle, pustule, umbilicated pustule, scab, desquamation. Lesions were resolved by day 18, with the exception of animal #3 (Figure 4A).

Confirmation of exposure and consistency of exposure between animals was performed on day 0 within 2 min of intravenous administration of the inoculum (Figure 4B). Animal #3 had roughly one log less circulating genomes after exposure, suggesting that not all of the inoculum was received intravenously. Although normalized to the limit of detection (in Figure 4B), levels in the three animals fell well below the limit of detection and sharply rebounded in all animals on Day 2, and peaked between days 8 and 10. Surprisingly, examination by plaque assay titration revealed no infectious virus during this period. Select chemistry and hematology results are also presented as (Supplemental Data Figure S1).

#### 3.3.3. Postmortem, Microscopic, and Immunohistochemical Findings

Two of the three EV-exposed animals (Animals #1 and #2) survived to the study endpoint and were humanely euthanized on day 30 post-exposure (approximately 12 days after disease resolution). The third animal (Animal #3) was humanely euthanized on day 18 post-exposure due to a decline in clinical condition and was not considered moribund due to poxvirus infection. Because this animal’s disease course was not typical, it will be discussed separately.

Gross, histopathologic and immunohistochemical findings in Animals #1 and #2 were reflective of a mild infection, with virus dissemination post-exposure and extensive viral clearance as evidenced by little to no poxviral antigen staining in target tissues at day 30 post-exposure. No MPXV-related macroscopic observations were noted in the lungs from either of the two surviving monkeys. Microscopic pulmonary findings typical of MPXV infection were noted in Animal #2, characterized by subpleural necrotizing and lesions that were immunopositive for MPXV antigen. Cutaneous macroscopic observations consisted of desquamated scars on the face, arms, legs, axillary and inguinal regions, and/or abdomen. Microscopically, cutaneous lesions were characterized by histiocytic inflammation and epithelial hyperplasia. Enlarged axillary and/or inguinal lymph nodes were observed macroscopically in both surviving monkeys. Microscopically, lymphoid hyperplasia was noted in multiple lymphoid organs; this was considered to be a nonspecific finding reflecting antigenic stimulation. All remaining tissues from the two monkeys euthanized on day 30 post-exposure were immunonegative for MPXV antigen.

Animal #3 was humanely euthanized on day 18 post-exposure due to necrosis and ulceration in the hind limb attributed to extravasation of the challenge inoculum. Cutaneous lesions consistent with monkeypox infection were widely disseminated in inguinal and axillary areas, lips, chest, abdomen, arms, legs, and particularly striking over the affected right hind limb. Microscopically, cutaneous lesions were characterized by histiocytic inflammation, necrosis, and epithelial degeneration with intracytoplasmic inclusion bodies typical of poxviral inclusions. A solitary 3–4 mm pale nodule was observed on the pleural surface of the lungs. Microscopically, the nodule was characterized by a focal necrotizing and proliferative lesion, typical of MPXV infection. Additional microscopic findings attributed to EV infection consisted of necrotizing and proliferative steatitis surrounding the mesenteric lymph node. Immunohistochemistry revealed extensive immunoreactivity of macrophages and fibroblasts in the lung lesion, skin and underlying skeletal muscle of the affected hind limb, and perinodal mesenteric adipose tissue. Minimal immunoreactivity was detected in macrophages in lymphoid tissues. Circulating leukocytes were not immunoreactive.

### 3.4. Stability of EV in Nonhuman Primate Sera

**Figure 5:**
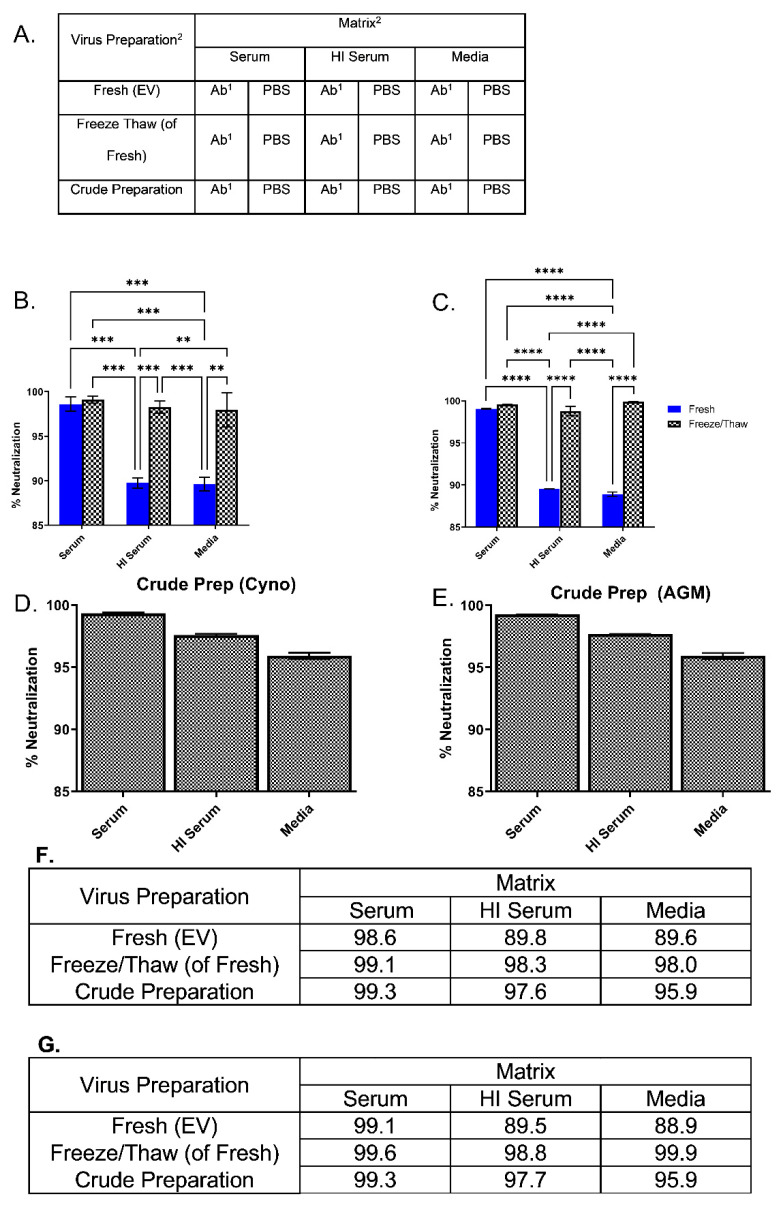
Effect of nonhuman primate sera on MV neutralization of freshly prepared and freeze/thawed preparations of monkeypox virus. (**A**) A general outline of the experimental design. (**B**) Fresh preparations of monkeypox virus were split and incubated with either cynomolgus (Cyno) serum or HI serum and anti-L1 ab (7D11) or PBS (n = 3). (**C**) This was repeated with AGM serum (n = 2). A portion of these samples were freeze/thaw/sonicated before the addition of serum, antibody, or PBS control (**B**,**C**). (**D**,**E**) Our animal virus stock (crude stock) that has undergone multiple freeze/thaw and sonication cycles was treated similarly, n = 3. Statistical differences, using a two-way ANOVA and Sidak’s multiple comparisons (Graphpad Prism), were noted (**** *p* < 0.0001, *** *p* < 0.001 and ** *p* < 0.01). Consolidated table of neutralization values are given for cynomolgus macaque sera (**F**) and AGM sera (**G**). There was no statistical difference between serum, crude, and any of the freeze thaw groups (*p* > 0.05). There was also no statistical difference between analogous cynomolgus macaque and AGM treatment groups (*p* > 0.05).

Since no discernible change in virulence was noted in the animal experiments, we questioned whether this was because the EV had no additional role during intravenous exposure, or if the route of exposure influenced the morphogenic state of the virus. Based on the literature, where EVs were protected from the effect of host-homologous complement, our initial hypothesis (pre-animal exposure) was that the effects of serum would minimally impact the integrity or infectivity of the extracellular virus [34]. Preliminary investigation involved incubating a fresh preparation (analogous to enriched inoculum preparation) with either heat-inactivated or fresh cynomolgus macaque sera. Anti-MV antibody, MAb-10F5, was incorporated in both preparations. In the context of fresh cynomolgus macaque serum, neutralization of monkeypox virus increased, suggesting disruption of the EV. To further test and add appropriate controls (Figure 5A), we freshly prepared monkeypox virus and collected the EV enriched supernatant using the same methodology used for the preparation of the inoculum used in the animal challenges. The clarified supernatant was then incubated with NHP serum, heat-inactivated NHP serum, or PBS, followed by the addition of anti-MV antibody (7D11) to eliminate MV, or PBS as a control. Aliquots of the fresh preparations were freeze/thawed/sonicated and treated similarly. This process was intended to disrupt the EV, resulting in MV-like particles and showing that the neutralized particle was indeed protected, and that neutralization was not a product of suboptimal concentration of antibody.

To show that the effect was specific to the fresh preparation (EV), crude stock virus was also tested concomitantly (Figure 5). All samples were analyzed by plaque assay. Initially, cynomolgus macaque sera were tested, as this was the host species for the animal experiment. In the context of the fresh preparation, the ability of 7D11 to neutralize MV increased from approximately 90% when heat-inactivated serum or PBS was used to 99% for serum (Figure 5B). By freezing, thawing, and sonicating, thereby disrupting the outer membrane, the neutralization increased to statistically comparable levels (*p* > 0.05). The crude preparation did not differ greatly from the freeze/thawed material (*p* > 0.05) and ranged from 98% to 99% (Figure 5D). Since the virus was prepared in an AGM kidney cell line (Vero-E6), we wanted to ensure that this neutralization was not a function of the host complement and the cell line derived outer membrane being nonhomologous [10]. In a mirrored experiment, AGM serum was evaluated and the results were similar in analogous groups (Figure 5C,E). It is questionable whether or not this increased MV neutralization is due to serum alone since titers remained relatively constant between groups. These data suggest that virions not susceptible to MV-neutralizing antibodies (i.e., EV) become susceptible after treatment with NHP serum from either AGMs or cynomolgus macaques.

**Figure 6:**
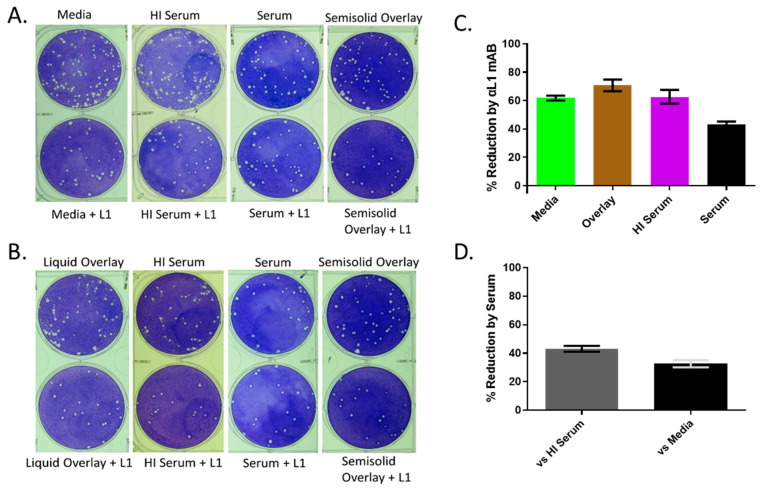
Effect of serum on monkeypox virus comet formation. (**A**,**B**) Two concentrations of monkeypox virus were adsorbed on Vero E6 cells for 2 h and unattached virus was subsequently removed by washing. A final concentration of 10% cynomolgus serum or heat-inactivated serum in the presence or absence of anti-L1 antibody (1:500) was used to overlay the cells and a 1.5% methylcellulose overlay with and without anti-L1 antibody was utilized as a no-comet control and to determine the effect the antibody on the primary plaques. (**C**) Percent primary plaque reduction in the presence of anti-L1 antibody within each matrix listed on the *X* axis. (**D**) Percent primary plaque reduction of serum using either the heat-inactivated serum or media to normalize. High virus concentration was a target of 100 pfu/well, whereas intermediate virus concentration was a target of 50 pfu/well.

To further illustrate this point, comet inhibition assays were performed (Figure 6). Extracellular virus released from the cell forms satellite or comet plaques. In the presence of cynomolgus macaque or African Green monkey (data not shown) serum, the number of comets was drastically reduced (Figure 6A,B), but not if the serum was heat-inactivated. When MAb 7D11 was added, the reduction was even more apparent. In fact, MAb 7D11 had anti-comet activity by itself as exemplified when heat-inactivated serum or media alone was present (Figure 6C). From these data, it is likely not all the EV produced are intact and, therefore, are susceptible to a heat labile component of the serum (e.g., complement) or an anti-MV antibody capable of inactivating the underlying MV. It is important to note that the addition of serum or MAb 7D11 also reduced the number of primary plaques present by 65% and 43% respectively (Figure 6C,D). This reduction by serum could theoretically reduce the number of secondary plaques (comets) in the assay. To account for this, we reduced the viral input for the comet assay by 50% and 75% (Figure 6B). In comparison, the addition of serum still qualitatively produced less satellite plaques. The combination of these assays provides strong evidence that the monkeypox EV administered via the intravenous route does not remain intact. This suggests either a technical hurdle in producing the proper EV for dissemination, or monkeypox EVs are not stable whether through natural infection or after experimental infection.

### 3.5. Detection of EV in Oral Swabs

**Figure 7:**
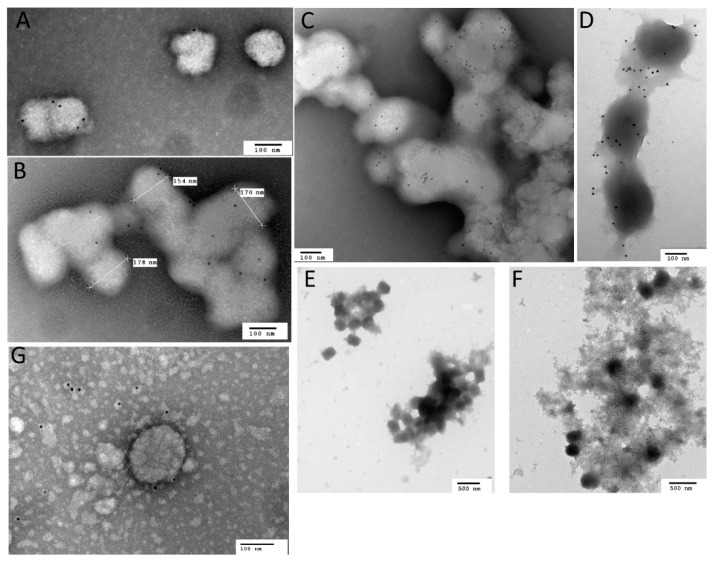
IEM of EV detected in oropharyngeal swabs of intravenously infected macaques. Oral swabs were collected three days post-exposure (**A**–**C**,**G**) or cell culture (**D**–**F**) with monkeypox virus prepared and stained with antibody against EV (**A**–**E**,**G**) or MV (**F**) for IEM. Cell cultures of monkeypox virus were either prepared with (**D**,**E**) or without (**F**) purification. Measurements of individual virions are given for (**B)**. For (**A**–**D**,**G**), scale bars are 100 nM; 500 nM for (**E**,**F**).

Next, we questioned whether EV could play a role in transmission by addressing whether EVs were present in oral samples. Oral swabs were collected from two nonhuman primates three days after intravenous exposure to the MV form of monkeypox virus. Samples were collected and examined by IEM to detect the EV forms of monkeypox virus. In one animal, particles were present that labeled with anti-EV antibody, 10F10, to varying degrees (Figure 7A). From the same sample, there were also multiple instances of labeled virions within a uniform structure (EV bundles) (Figure 7B,C). We compared these findings with cell culture preparations of monkeypox virus that were purified by sucrose cushion followed by a sucrose gradient. The upper band, typically corresponding to EV, contained features similar to what we detected in oropharyngeal swabs (Figure 7D,E). As a representation of debris and how it compares to these bundles, we also looked at a crude preparation of monkeypox virus stained with an anti-MV monoclonal antibody (Figure 7F). Although we found membrane-like material surrounding virions, the morphology was different, as the material looked unstructured with few MV (or MV-like particles) present and most likely represented debris. In the other animal, few virions were present and were unconvincingly labeled, and likely were more MV-like (Figure 7G).

## 4. Discussion

One of the major drawbacks to the monkeypox intravenous macaque model is the unnatural quantity of virus required to produce severe disease. Although other routes of exposure can reduce the input of virus required for lethality, ≥5 logs of virus are still needed and skin lesions are reduced [27]. Therefore, one can conclude that macaques are innately resilient to severe disease induced by monkeypox virus or that there are other experimental factors that have yet to be explored. Of these factors, we found it worthy to expand on the properties of the inoculum. We optimized the matrix (constituents of the inoculum) and found that purification of the virus did not increase virulence in macaques, and that the use of non-purified virus led to more consistent endpoints, most likely due to aggregation of the purified stocks [29]. In our current study, we focused on the morphological makeup of the virus used for exposure. Historically, the MV form of monkeypox virus (and other orthopoxviruses for alternative models) has been utilized. Here we report our first attempt to infect animals with a preparation that includes the extracellular form of an orthopoxvirus.

The envelope of the extracellular virus is thought to be more fragile than that of the intracellular mature virus and purification, freeze/thaw cycles, and sonication are known to disrupt the membrane [34]. Additionally, the variability/repeatability of purifying virus, as well as difficulties accurately titrating purified stocks [29] would necessitate plaque titrating before challenging the animals. Because of this added step, stocks would require approximately 4–6 days in the refrigerator and risk additional membrane degradation. Given the technical complexities, researchers have typically used fresh preparations for in vitro experiments requiring the extracellular form, but these preparations contain contaminating MV, which are typically neutralized using MV specific antibodies [9]. Given the downsides of purification, we chose to use fresh preparations.

Here, we characterized the morphogenic makeup of a fresh preparation of monkeypox virus propagated for 24 h in the Vero E6 cell line at a high MOI. Experiments at 24 and 48 h using MOIs of 3, 10, and 30 were also conducted and, with the exception of virus propagated for 24 h at an MOI of 3 (96%), neutralization was between 88% and 91%, similar to those used for further experimentation and animal exposure. Sonication and freeze/thaw cycles liberated the enclosed MV-like particle increasing the neutralization to 97%–99%, but never 100%. Immunoelectron microscopy confirmed the presence of EV in the fresh preparation and the relative absence of EV from our historical stock. These data indicate that approximately 7%–9% of viruses in the inocula were EV with intact outer membranes. Based on the results of our comet assay, where comets were reduced in the presence of MV-neutralizing antibody, it is likely that more EV may be present, but lack outer membrane integrity. This would explain the initial high neutralization of the fresh preparations (approximately 90%), where the extracellular form of the virus should be prevalent, as exhibited by our comet assays (Figure 6). Ma et al. had similar findings when ectromelia virus was incubated with anti-L1 polyclonal or certain monoclonal antibodies [37]. Reports for the EV content of vaccinia virus have ranged from 10% to 40% up to approximately 71% [34,38,39,40,41]. Our results are more reflective of those proposed by Schmidt et al., although with less variability, and those we have previously reported for vaccinia strain WR [9]. The variability seen in the Schmidt studies may be a function of a low MOI in conjunction with time. For instance, we found that monkeypox virus inoculated at MOI of 3 was reduced by 96% after 24 h but only 88% after 48 h (data not shown). Furthermore, we had greater variability regarding total virus output at 24 h at lower MOIs. In any case, a head-to-head comparison between orthopox virus utilizing similar reagents, cells, and experimental conditions should be performed in order to evaluate any true differences between MPXV and other orthopoxviruses.

We were unable to obtain our target titer of approximately 5 × 10^6^ pfu/mL for our inoculum, a dose that could cause a severe but not uniformly lethal disease in NHPs. We chose to continue the experiment and rationalized that the properties of EV could still make a significant difference while only comprising 7%–9% of the inoculum; in essence, we theorized that the small proportion of EV in the inoculum could disproportionately affect infection and disease. In addition to resistance to complement, EV have also been shown to have faster binding and entry kinetics [13,42,43] and are approximately 5 times more infectious [13,38,43]. Theoretically, complement resistance and efficiency of EV infection could sufficiently overcome the dosing deficit and increase the intensity of disease (e.g., death, lesion counts) relative to a similar dose, 3×10^5^ pfu/mL, of MV [26]. This is possible given the steep dose response inherent to the intravenous monkeypox model ([26] and reviewed by [27]).

We next tested whether or not expunging the supernatant through a syringe would damage the EV outer envelope. We found that the conditions used to produce the inoculum for exposure were repeatable and the mechanical stress induced by mimicking an intravenous exposure did not affect the integrity of the EV membrane, as determined by the MV-neutralizing assay.

Exposure of cynomolgus macaques with similar material produced a mild smallpox-like disease. All animals survived the challenge, although Animal #3 was humanely euthanized prior to study termination for other reasons. Of the remaining animals, cutaneous lesions were few (64 and 203 lesions) and appeared concomitantly with lymphadenopathy on day 6. Although increases in temperature were noted, and interestingly biphasic in one case, a febrile state, as defined by a 2 °C increase from baseline, was not achieved in either animal. These findings are comparable to cynomolgus macaques exposed to crude preparations of monkeypox virus at a similar dose [26], but much less severe than the 5 × 10^7^ pfu intravenous model. With that being said, the onset and magnitude of viral DNAemia was strikingly similar to the 5 × 10^7^ pfu model. This is despite two of the animals that received the entire inoculum intravenously (Animals #1 and #2) having qPCR levels far below the limit of detection. The magnitude of the viremia was greater (2 log_10_) and peaked later than reported results using traditionally prepared virus at a similar dose. Interestingly, no viable virus was detected from whole blood samples during the course of infection. It is possible that virus contained within the blood samples at early time points were not detectable due to the inherently high limit of detection when assaying blood samples. As for subsequent samples, it has been shown that animals exposed to similar doses of crude virus develop neutralizing antibody as early as day 5 [26]. Interestingly, animals exposed to EV preparations had delayed increases of circulating WBC relative to both doses of purified material. The reason for this is unknown, but it could relate to other viral modulators present in the preparation or some other properties imparted by a partially intact EV (or membrane component). Although these findings are interesting and useful for fostering future research, they are anecdotal in nature and would require additional experiments to confirm.

We examined whether EV could have been responsible for the small changes exhibited in vivo. From our MV specific neutralization assays, we found that freshly prepared EV became susceptible to MAb-7D11 in the presence of 50% serum, but not heat-inactivated serum, obtained from two nonhuman primate species. Furthermore, it required freeze/thaw fracturing to allow the MV-neutralizing antibody to affect the infectivity of the remaining (roughly 8%) EV. These results were confirmed by comet assay where secondary plaque formation (EV spread) was drastically reduced in the presence of 10% nonhuman primate serum. The addition of MAb 7D11 further reduced the presence of comets, as well. By itself, the comet assay suggests that serum and MAb 7D11 might work by the same mechanism, most likely taking advantage of ruptured EV, since MAb 7D11 also dramatically reduced comet formation. Some caution must be taken when examining mechanism of EV neutralization, as CEV and EEV could be different [9]. The data from the neutralization assays suggest that most, if not all, EVs are converted to the underlying MV form and neutralized, as comparisons between samples in which the outer membrane was stressed (freeze/thaw/sonication) and crude preparations that were shown to be MV (at the limit of detection of IEM) produced similar results. Together, these data suggest a mechanism by which the serum, or some heat labile component thereof (likely complement) may rupture the membrane of the remaining intact EV allowing neutralization by complement and/or MAb-7D11. A similar mechanism has been proposed by Lustig et al. in which anti-A33 antibodies activate complement to lyse the outer membrane of the EV allowing neutralization by 7D11 [38].

Although the experiments presented in our report cannot completely differentiate the mechanism or define the exact membrane status of the viral particle, they provide evidence that few, if any, intact EVs were effectively administered to the cynomolgus macaques. Our results also question whether the morphological constituents of monkeypox viremia could be of the extracellular form or if we may be utilizing the wrong cell lines/methodology. Vero cells have been shown to have the regulators of complement activation (RCA) thought to provide resistance [39]. We did not ask whether or not monkeypox EV formed from Vero cells bare these RCA, nor did we enrich the host-specific RCA content in the EV by testing multiple cells lines. These factors were proposed potential mechanisms for EV complement resistance. It is possible that in vivo, EV may be stably produced depending on the host cell. Additionally, other stabilizing factor(s) absent from our in vitro test system could also contribute. Another approach to answer this question would be to ask which viral forms are present during the viremic phase of this (or any model), similar to Payne et al. [20]. For monkeypox virus in nonhuman primates, a non-intravenous, non-macaque model would be preferred to answer such a question since the exposure itself represents the “secondary viremia” phase and would convolute the interpretation of data. Now that such models exist, as well as better, more sensitive and specific antibodies and assays [9], future studies will look at the composition of the viremia.

Similar to other reports with vaccinia virus, MAb-7D11 neutralized monkeypox virus [42,43,44]. Interestingly, it also reduced primary plaque formation post-adsorption (2 h at 37 °C), similar to reports with vaccinia virus [9]. It has been shown with vaccinia virus that L1 is necessary for entry into the host cell, as cores from virions lacking L1 could not penetrate the cellular membrane and that addition of soluble L1 can block entry of vaccinia virus, suggesting a role in entry [45]. Additionally, MAb-7D11 has been shown to neutralize vaccinia virus under conditions that would suggest a post-attachment but pre-entry step [43,45]. Our data suggest that, in the context of our test system, MAb-7D11 can either neutralize post-entry (in the endosome) and/or a portion of the monkeypox virus remains bound to the cell surface for an inordinate amount of time, allowing neutralization (and subsequent plaque reduction). The latter explanation would support findings by Bisht, Weisberg, and Moss and Foo et al. and also explain our novel observation of neutralization of primary plaques by serum [45,46].

It is unclear what morphogenic form of the virus is naturally transmitted from host-to-host, but poxvirus canon dictates that EVs are too fragile to manage this task. One must consider that if transmission of MPXV and VARV is by direct contact (via respiratory droplets), it would logically follow that robust attributes are not required for the nominal period outside of the host. Moreover, the viral membrane may be stabilized by components in the extracellular environment. Additionally, it should be asked which particle is more capable of infection. Relative to MV, EV infection is kinetically more favorable [13,38,42], relatively resistant to complement [10] and antibody neutralization [11,12]. These characteristics are not only advantageous for initial infection, but also for prolonged shedding despite host response [47,48]. Payne and Kristensson provided evidence for the presence of EV as the major form of virus in the oropharyngeal region of mice infected via the intranasal route with cowpox virus [24]. Similarly, we observed the presence of EV in the oral cavity (oropharyngeal swabs) in the lesional (intravenous) NHP model on Day 3. In this model, seeding of the respiratory tract and subsequent shedding is a result of the systemic challenge of virus. Since no EVs were used to infect these animals, our detection of EV in throat swabs suggests that there was productive infection after exposure. Unlike Payne and Kristensson, the production of EV from a specific area/tissue was not determined, as this would have required euthanizing to obtain the proper tissue samples. Although the respiratory macaque model is probably not sensitive enough (e.g., unfavorable virus dose response) to determine whether monkeypox virus EVs are relatively more infectious than our typical MV preparations, the question should be asked using a more sensitive model, such as the marmoset model [3,4] or the Cast EiJ mice [49].

More studies should be conducted to inquire if the EV phenomena (aggregated EV bundles) present in the oral samples have some role in transmission or are just an artifact. If real, the aggregation of monkeypox virus may be the reason for inefficient respiratory transmission and/or be a mechanism for environmental stability, possibly similar in function to A-type inclusion bodies produced by some poxviruses [50,51,52]. In Mucker et al., we provide ancillary evidence that aggregation can be an issue with intravenous injection. In those studies, purified monkeypox virus (MV) administered IV had characteristics of an aggregated virus preparation and the ensuing disease was more inconsistent ([29]. Furthermore, in the studies presented here, we were unable to completely neutralize unpurified MV with MAb 7D11, regardless of dilution of the monkeypox virus. This may be related to aggregation of MV (e.g., blocking epitopes), as other poxvirus can be completely neutralized by MAb 7D11 [9], but more studies are needed. Our hope is that these data could help shape future studies to test the contribution of EV in transmission. Studies to look at the kinetics of the morphogenic forms of multiple orthopox viruses over time, as well as infection studies, could provide key insights into transmission.

## Data Availability

Data is contained within the article or Mucker et al. [29].

## Supplementary Materials

The following supporting information can be downloaded at: https://www.mdpi.com/article/10.3390/v14091993/s1, Figure S1. Select hematology and albumin levels of animals exposed to an EEV enriched preparation of monkeypox virus.
