# Supplementary material for: Evaluation of Virulence in Cynomolgus Macaques Using a Virus Preparation Enriched for the Extracellular Form of Monkeypox Virus
Source: Viruses. 2022 Sep 9;14(9):1993. doi: 10.3390/v14091993 (PMC9505131; doi:10.3390/v14091993)
Supplement: Supplementary file 1 [file viruses-14-01993-s001.zip › viruses-1879874-supplementary.pdf]

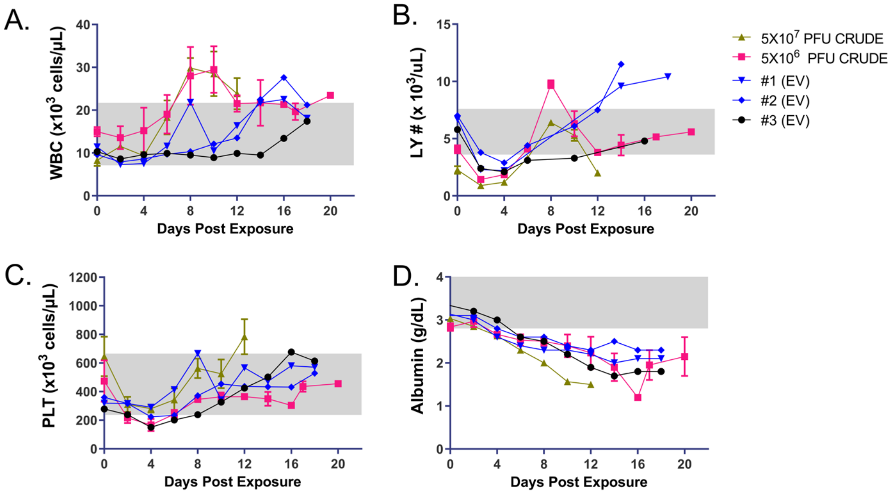

Figure S1. Select hematology and albumin levels of animals exposed to an EEV enriched preparation of monkeypox virus. Hematology using EDTA whole blood was performed on a Coulter AcT10. Chemistry was performed using a General Chemistry Panel on Piccolo analyzers. (A) White blood cells (WBC); (B) number of lymphocytes (LY #); (C) platelets (PLT); (D) albumin. Reference ranges (grey boxes) were provided by USAMRIID with the exception of WBC [32].
